# Is resistance futile? Life-history costs of escaping parasitoid attack in a major crop pest

**DOI:** 10.1093/jee/toaf338

**Published:** 2025-12-05

**Authors:** Mia C McGowan, Rebecca A Boulton

**Affiliations:** Biological and Environmental Sciences, Faculty of Natural Sciences, University of Stirling, Stirling, UK; Science and Advice for Scottish Agriculture, Edinburgh, UK; Biological and Environmental Sciences, Faculty of Natural Sciences, University of Stirling, Stirling, UK

**Keywords:** biocontrol, resistance evolution, whitefly, parasitoid, heritability

## Abstract

Resistance evolution occurs when genetic variants that confer increased survival in the face of mortality causing factors (ie pathogens, parasites, and predators) increase in frequency within a population. It has profoundly shaped life on earth, but in the Anthropocene its implications for human health (antimicrobial resistance) and food security (pesticide resistance) are more widely discussed. Inegrated Pest Management strategies which integrate chemical control with the use of evolvable natural enemies such as parasitoid wasps have been designed to minimize resistance evolution in pests and protect food security. However, in recent years there have been more reports of pests becoming resistant to their biocontrol agents. In this study we investigate a potential emerging example of resistance to biocontrol in the glasshouse whitefly, *Trialeurodes vaporariorum* (Hemiptera: Aleyrodidae) Westwood, to the parasitoid wasp *Encarsia formosa* (Hymenoptera: Aphelinidae) Gahan. We found evidence for genetic variation in the whiteflies ability to survive parasitism based on quantitative genetic estimates of narrow-sense heritability derived from a family-level survival experiment. In a second experiment, we show that surviving parasitism attempts by *Encarsia* is costly for *Trialeurodes*; whitefly that were attacked and survived had lower fecundity and their eggs were less likely to successfully hatch compared to unexposed controls. We discuss the ways in which this life history trade-off between survival and reproduction when exposed to *E. formosa* could be exploited, for example by intercropping with suboptimal host plants, to maintain susceptible genotypes and slow resistance evolution.

## Introduction

Resistance evolution occurs when organisms evolve adaptations that increase their survival in the face of mortality-causing factors. If a proportion of the population harbors genetic variants that increase their survival in the face of the mortality-causing factor, then they will survive and reproduce; individuals without these beneficial alleles will not. This results in the rapid spread of alleles that confer resistance and ultimately populations that are resistant to pathogens, parasites and other lethal factors. Resistance evolution is now a major threat to human health and food security due antimicrobial resistance and pesticide resistance respectively (Popp et al. 2013). The evolution of resistance to traditional chemical pest control has been widely demonstrated and the problems it causes for food security are well-documented (see Pimentel 2005).

Biological control agents are natural enemies of pests and include pathogens, parasitoids, and predators, and in some cases inert products (ie toxins) derived from these living organisms (Heimpel and Mills 2017). Biological control agents can have a significant advantage over chemical control; as evolutionary entities themselves, they can, in theory, co-evolve alongside the pests that they are used to control, maintaining their efficacy in the face of resistance evolution (Holt and ­Hochberg 1997).

However, in the case of augmentative biological control (where commercially reared natural enemies are released in large numbers to control the pest population; Heimpel and Mills 2017, Hajek 2018), this theoretical advantage may be diminished. This is because commercial rearing of biocontrol agents often involves breeding them using hosts which are highly susceptible to their natural enemies (for sensible logistical reasons). As a result, parasitoids can experience relaxed selection for traits that allow them to overcome their hosts defenses (virulence traits; Coelho et al. 2016). Moreover, lab adaptation and drift can result in the loss of genetic variation underlying these virulence traits, limiting parasitoids’ effectiveness in biocontrol and ultimately their ability to mount a coevolutionary response (Coelho et al. 2016). Pest populations, on the other hand, are exposed to their natural enemies and so experience selection to overcome parasitism, making pest resistance more likely to evolve than parasitoid virulence. This could give pests an evolutionary advantage over their biocontrol agents, potentially undermining the efficacy of augmentative biocontrol.

Formal research on the evolution of resistance to biocontrol has not been as extensive as research on chemical pesticide resistance, but there are now several examples where parasitoid-based biocontrol has broken down due to pest resistance (ie Goldson et al. 2014, Ives et al. 2020, Beekman et al. 2022, Shields et al. 2022). This literature includes a substantial body of evidence that demonstrates how endosymbiont bacteria confer resistance to parasitoid attacks in a variety of common pest aphids (ie Käch et al. 2018, Beekman et al. 2022, Vorburger 2022).

In this study, we examine the potential for resistance evolution in another pest, the glasshouse whitefly (*Trialeurodes vaporariorum*). This species is an arrhenotokous haplodiploid (Las 1980) in the Hemiptera, but unlike its aphid relatives there is no evidence that endosymbiont bacteria protect it from parasitoids (Andreason et al. 2020). However, our pilot observations indicate that some individuals can survive parasitism attempts, and anecdotal reports from growers suggest that the efficacy of its primary biocontrol agent, the solitary endoparasitoid *E. formosa*, can decline over the growing season (M. Graham, British Tomato Growers Conference 2019, pers comm). Together, these observations point to the possibility that whiteflies harbor genetic variation which could enable them to evolve resistance to *E. formosa*.

*T. vaporariorum* infest over 800 plant species, including tomatoes, strawberries and ornamentals (Byrne and Bellows 1991, Hoddle et al. 1998) causing significant reductions in yield through direct (feeding) and indirect (pathogen transmission) damage(Byrne and Bellows 1991, McKee et al. 2007). Whitefly have readily evolved resistance to both old (Wardlow et al. 1976) and new (Karatolos et al. 2012, Ilias et al. 2015) classes of pesticides, and as a result, biocontrol with *E. formosa* has been vital for protecting crops from whitefly since the 1970s. As such, the anecdotal reports from tomato and soft fruit growers in the United Kingdom (M. Graham, British Tomato Growers Conference 2019, pers comm) that *E. formosa* loses effectiveness over the growing season are troubling. While biocontrol failures of *E. formosa* could be increasing due to changing environmental conditions (ie higher humidity, Ekbom 1977, Milenovic et al. 2023), the geographically widespread nature of the reports hint at resistance evolution could also be involved (ie Postic et al. 2020, Beekman et al. 2022).

Our aim in the current study was to quantify variation in survival after *E. formosa* parasitism in the glasshouse whitefly *T. vaporariorum*. We found that across families of whitefly there was substantial variation in survival after parasitism by *E. formosa*, suggesting the potential for resistance evolution in this system. However, in order to evolve, individuals which survive a parasitism attempt must successfully reproduce. Life history trade-offs are ubiquitous in nature as all individuals only have a finite amount of resources to invest in survival and reproduction (Gwynn et al. 2005). As such, whitefly which survive parasitism are expected to have reduced reproductive success compared to unparasitized controls (Holt and Hochberg 1997), a prediction we tested in a follow-up experiment.

## Materials and Methods

### Experimental Organisms and Rearing Conditions

We used a laboratory colony of *T. vaporariorum* (hereafter ‘whitefly’) originally sourced by Bioline Agrosciences from multiple UK sites. The colony (∼4,000 founders) was maintained on black beauty aubergine (*Solanum melongena*) plants in BugDorm^®^ cages at the University of Stirling Controlled Environment Facility (26 °C, 70% RH, 16:8 L: D cycle). Plants were watered every 2 days and fertilized weekly with Tomorite (Levington, United Kingdom). The parasitoid *Encarsia formosa* was obtained as pupae from the same supplier.

### Quantifying Variation in Resistance

From the main whitefly colony, we generated 35 distinct full-sib families by pairing (see Supplementary Fig. S1 and Supplementary Table S1 for crossing design). These families were generated by takingfreshly-eclosed virgin F0 adults from the main colony (adult whitefly do no mate immediately on emergence, collecting adults within an hour of eclosing ensured virginity, Perring et al. 2018). F0 whitefly were paired (1 male, 1 female) and put on isolated aubergine leaves in clip-cages (Supplementary Fig. S2) to produce the F1 generation. Unrelated F1 individuals were then paired (*N* = 35 pairs) to produce the F2 generation which we used for resistance assays. These clutches were used to establish family-matched F2 replicates that were either exposed to *Encarsia* (treatment) or unexposed (control).

When 2nd instar nymphs appeared in the F2 clip cages (around ∼ 10 days after laying), *E. formosa* were applied to treatment groups at a 1:5 wasp-to-nymph ratio (nymphs were counted before exposure) for 48 hr. This design allowed us to directly compare survival across treatments within the same family genotypes. Survival outcomes (living, dead, parasitized nymphs, or emerged adults) were recorded daily for 10 days post-exposure.

### Measuring Life History Costs of Resistance

We infested thirty 10-week old aubergine plants in flowerpot cages (Watkins and Doncaster, UK) with 150 whitefly each. Adult whitefly were removed from the cages after 4 days, leaving only whitefly eggs which were left to develop for 10 days (second instar).

Of the 30 infested plants 15 were placed into a separate “control” cabinet which was free from *Encarsia*. The other 15 plants were treated with *E. formosa* (following the protocols outlined above under “*Quantifying variation in resistance*”).

All whitefly nymphs were checked for signs of parasitism after *Encarsia* removal using a hand lens. When nymphs are parasitized by *E. formosa*, a dark melanisation mark appears on the nymphal case (see Supplementary Fig. S3). Evidence from related systems suggests that these marks are reliable indicators of oviposition rather than non-reproductive probing or host feeding (Brady and White 2012). Any whitefly nymphs in the *Encarsia* treatment without a dark mark were removed from the plant using a paintbrush. All plants were left for 10 days for whitefly to complete development and eclose as adults.

### Experimental Set up

To collect focal whitefly which were virgins we checked plants for emerged adults every hour for 8 h.

We took 2 male and 2 female whitefly from each of the 30 plants (15 control and 15 *Encarsia*). One male and female from the same plant were placed together on a leaf disk (this represents one replicate). Leaf disks were 3 cm discs cut from fresh aubergine plant leaves and set in 1% agar in a 5 cm petri dish. Every 24 h for 4 days, or until the female died, the pair was moved to a fresh leaf disk. The number of eggs laid on each leaf disk was recorded. Four days after laying leaf disks were checked and the number of first instar nymphs that hatched was recorded. Hatching checks were repeated every 24 h for a further 4 days to quantify hatching success and hatching rate (we have observed previously that eggs which do not hatch after 4 days are unlikely to hatch at all).

### Data Analysis

#### Quantifying Variation in Resistance to Parasitism

We used a binomial generalized linear mixed model to test the effect of treatment (*Encarsia* exposure vs Control) on whitefly mortality at day 10. The outcome variable was the number of dead and surviving nymphs modeled as a two-vector response using the cbind function. The model included a fixed effect of treatment and random effects of block, plant, dam ID, and sire ID.

We also estimated narrow-sense heritability (*h*^2^) for survival following parasitism by running a linear mixed-effects model where the response variable was the proportion of dead individuals at 10 days post-exposure. The model was run separately for the *Encarsia* and control treatments, and included random effects of dam (F), sire (M), block, and plant to partition genetic and environmental variance components using restricted maximum likelihood. We extracted variance components following the approach outlined in Hansen et al. (2024). Because *T.s vaporariorum* is a haplodiploid species we then used these variance components to calculate heritability under the framework for haplodiploid organisms developed by Liu and Smith (2000) (see Supplementary Methods S4 for more details).

Liu and Smith (2000) assume the following: additive–dominance genetic model without epistasis, gene dosage compensation in haploid males, and an approximately Hardy–Weinberg population structure. We also note that Liu and Smith (2000) recommend performing a joint scaling test on data from multiple generations (P, F_1_, F_2_, etc.) to check that the additive–dominance model provides a good fit before interpreting variance components. However, because our design was a single-generation sib-analysis, we could not apply this test. As such, we our reported heritability estimates were calculated under the assumption of additive-dominance which we acknowledge as a limitation. In Liu and Smith’s (2000) model, variance among sires (haploid males) reflects additive genetic effects and is equal to half the additive genetic variance (*V*_A_), ie ${\mathrm{Var}}_{\mathrm{sire}}=V_{A}/2$. Variance among dams (diploid females) reflects a mixture of additive (*V*_A_) and dominance (*V*_D_) variance, as well as shared environmental effects (*V*_Ec_), ie ${\mathrm{Var}}_{\mathrm{dam}\;}=V_{A}/4+V_{D\;}/2+V_{Ec}$.

We estimated additive genetic variance based on this as: $V_{A}=2\;\times \;{\mathrm{Var}}_{\mathrm{sire}}$ and calculated narrow-sense heritability as:


$$h_{2\;}=\;\frac{V_{A}}{V_{P}}$$

Where $V_{P}$ is the total phenotypic variance calculated as the sum of all the variance components ($V_{P}=V_{A}+V_{D}+V_{e}$) where $V_{D}$(dominance variance) was estimated by rearranging the formula from Liu and Smith (2000) from:


$${\mathrm{Var}}_{\mathrm{dam}}=(V_{A})/4+(V_{D})/2+V_{Ec}$$

To:


$${\mathrm{Var}}_{\mathrm{dam}}=(V_{A})/4+(V_{D})/2+V_{Ec}$$

We assumed that common environmental effects shared within dams ($V_{Ec\;}$) were captured by the variance components for plant and block. This approach yields a haplodiploid-adjusted estimate of narrow-sense heritability based on additive genetic variance only and should be interpreted as an approximate measure of the potential for an evolutionary response to selection on resistance to parasitism. We acknowledge the use of ChatGTP-4 in assisting with mathematical conversions. All calculations were independently verified by a colleague with expertise in mathematical biology.

#### Measuring Life History Costs of Resistance

We tested whether exposure to *Encarsia* and successful resistance to parasitism influenced fecundity, hatching success, and hatching rate using generalized linear mixed models (GLMMs). We ran 3 sets of models, all of which included a random effect of replicate ID nested within plant ID. To test whether treatment with *Encarsia* influenced the number of eggs laid, we used a GLMM with a negative binomial distribution (to account for overdispersion), including main effects of treatment and disk (and their interaction). To test for a treatment effect on egg-hatching success we used a GLMM with a binomial error structure, modeled as a two-vector response [cbind(hatched, unhatched)]; this model also included an observation-level random effect to account for overdispersion (Harrison 2014). A third model tested whether the time taken for eggs to hatch varied across the two treatments or depending on what day the eggs were laid. This included the proportion of eggs hatching as a 2-vector response, fixed effects of treatment and hatching day (and their interaction), plus an observation-level random effect to handle overdispersion.

For both datasets, we used the R packages glmmTMB (Brooks et al. 2017) and lme4 (Bates et al. 2015) to run the models and DHARMa to run model diagnostics (Hartig 2024). We estimated the proportion of nymphs surviving and binomial confidence intervals using the function “binom_confint” in the R package binom (Dorai-Raj 2022) and ggsignif (Ahlmann-Eltze and Patil 2021) to conduct and visualize pairwise comparisons across treatments and days. Significance values are reported at α = 0.05.

## Results

### Variation in Resistance to *E. formosa*

**Fig. 1.**
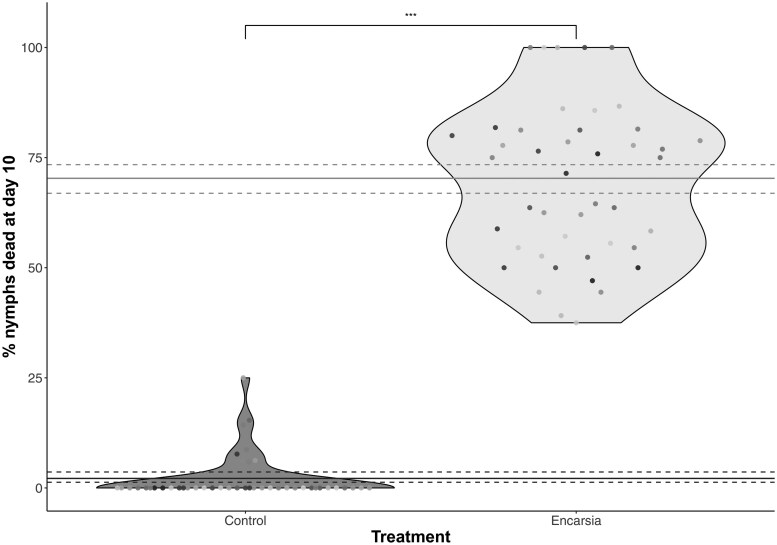
Percentage % of *Trialeurodes vaporariorum* nymphs which were dead 10 days after being exposed to the parasitoid wasp *Encarsia formosa* (Encarsia) or not (Control). Solid and dashed lines represent overall binomial mean ± binomial CI in the Control (bottom, dark grey) and *Encarsia* treated (top, light grey) whitefly. Survival was higher in the control whitefly compared to the *Encarsia* treated; significant differences are indicated by a * (<0.05) over the bars. Points on each plot are shaded by dam ID to aid with visual separation of families (*N* = 35 families; see Supplementary Fig. S4 for the same plot with points shaded by sire ID).

Across all families, around 30% of exposed whitefly nymphs exposed to *E. formosa* survived (compared to 97% survival in the control; effect of treatment $X_{1,86}^{2}$ = 248.88, *P* < 0.0001, Fig. 1). The proportion of nymphs that survived was highly variable across replicate families, some (*N* = 5 out of 35) suffered 100% mortality after *E. formosa* exposure, while in other families there was up to 60% survival (see Fig. 1).

Estimated narrow-sense heritability (adjusted for haplodiploid genetics) in the *Encarsia* treatment heritability was moderate (*h^2^* = 0.28). This value should be interpreted with caution, however, as the experimental design was not balanced across plants. Nevertheless, this value is useful in that it suggests a significant heritable genetic component to resistance to *Encarsia* (see Supplementary Figs S5 and S6 for a visual depiction of % survival across matrilines and patrilines). When we look at the variances in more detail, dam ID explained the highest % of variance in the model (45%), then sire ID (13%), then block (9%) and finally plant (6%; with 24% residual variance remaining).

We could not derive a reliable estimate of narrow-sense heritability (adjusted for haplodiploid genetics) for proportion survival in the *Control* treatment as mortality was extremely low, model fit was poor and there was insufficient variation to obtain a meaningful estimate.

### Life History Costs of Parasitism

**Fig. 2.**
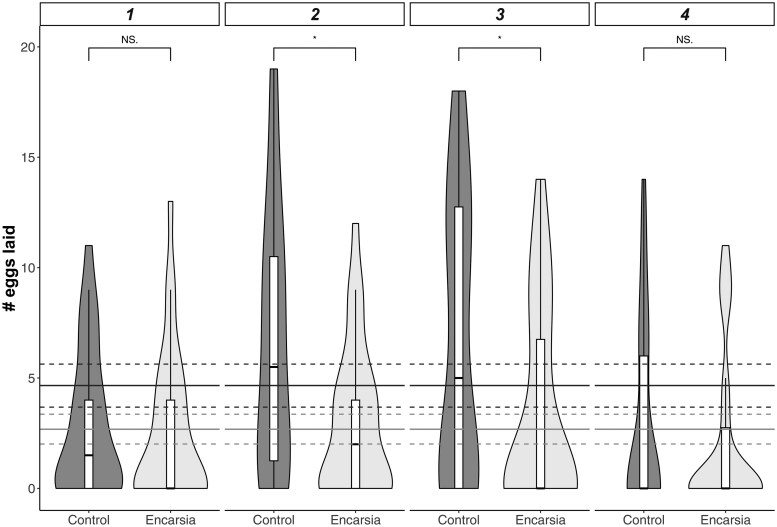
Number of eggs laid over 4 days by female *Trialeurodes vaporariorum* whitefly after being exposed and resisting parasitism by the wasp *Encarsia formosa* (*Encarsia*) or unexposed (Control). Solid and dashed lines represent overall mean ± 95% CI eggs laid per day across the 4 days by Control (top, dark grey) and *Encarsia* treated (bottom, light grey) whitefly. Overall whitefly which escaped parasitism laid significantly fewer eggs than control whitefly; significant differences in egg laying for each day are indicated by a * (<0.05) over the bars. Boxplots show the median (central line), interquartile range (IQR; box), whiskers extending to 1.5× the IQR, and individual points represent outliers beyond this range.

Exposure to parasitism by *E. formosa* reduced both egg production and hatch rate in *T. vaporariorum*. In terms of fecundity, whitefly that survived parasitism as nymphs laid on average 21.47 eggs (95% CI: 11.10–31.83) over 4 days, compared to 37.27 (95% CI: 29.31–45.22) for unexposed controls ($X_{1,28}^{2}$ = 4.16, *P *= 0.04; Fig. 2).

**Fig. 3.**
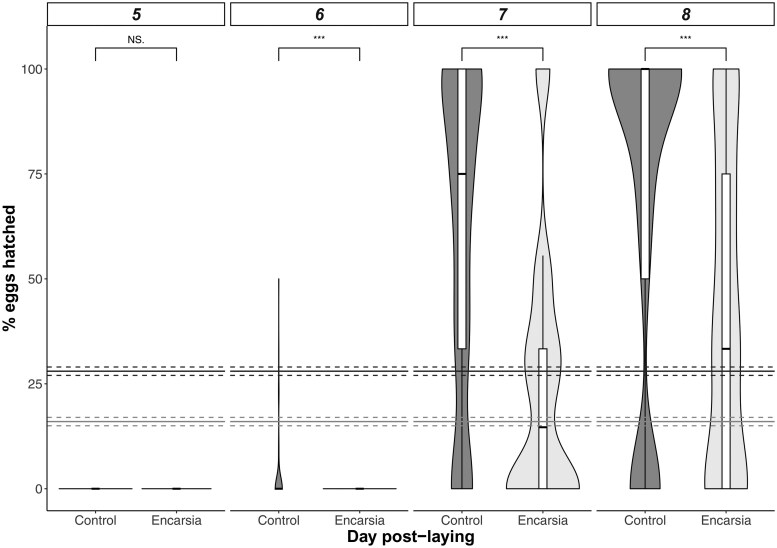
Percentage of eggs which hatched on days 4 to 7 post-laying by female *Trialeurodes vaporariorum* whitefly after being exposed to and resisting parasitism by the wasp *Encarsia formosa* (*Encarsia*) or not (Control). Solid and dashed lines represent overall binomial mean ± binomial CI in the Control (top, dark grey) and *Encarsia* treated (bottom, light grey) whitefly. Hatching success was higher in the control whitefly compared to the *Encarsia* treated; significant differences in hatching success for each day are indicated by *** over the bars. Boxplots show the median (central line), interquartile range (IQR; box), whiskers extending to 1.5× the IQR, and individual points represent outliers beyond this range.

In terms of hatch rate, 28.3% of eggs (binomial CI: 27.3-29.3) from control females successfully hatched in 4 days, compared to 16% (binomial CI: 15.0–17.1) for *Encarsia* treated females (treatment effect: $X_{1,28}^{2}$ = 8.81, *P* = 0.002; Fig. 3). Egg production peaked on days 2 and 3 in both groups ($X_{3,26}^{2}$ = 20.36, *P *< 0.001; treatment*day: $X_{3,26}^{2}$ = 3.10, *P *= 0.38; Fig. 2). Hatch rate did not vary with the day on which eggs were laid (main effect of disc: $X_{3,26}^{2}$ = 1.48, *P *= 0.69; disc*treatment: $X_{3,26}^{2}$ = 1.63, *P *= 0.65), and most eggs hatched on days 7 and 8 post-laying in both treatments (main effect of day: $X_{3,26}^{2}$ = 511.97, *P *< 0.0001; treatment × day: $X_{3,26}^{2}$ = 2.05, *P *= 0.56; Fig. 3).

## Discussion

The evolution of resistance to chemical pesticides poses a major threat to food security and is well documented across insect pests (Ilias et al. 2015) including the glasshouse whitefly, *T. vaporariorum* (Wardlow et al. 1976, Karatolos et al. 2012). In contrast, resistance to insect biological control agents has only been reported on a handful of occasions (eg see Hufbauer 2001, Goldson et al. 2014, Aya et al. 2019, Beekman et al. 2022, Shields et al. 2022, Vorburger 2022). One explanation is that host–parasitoid coevolution buffers against resistance evolution (Holt and Hochberg 1997). Yet, commercial rearing of biocontrol agents may reduce genetic diversity through drift and lab adaptation (de Paula et al. 2023), potentially tipping the evolutionary balance in favor of pests. Our findings suggest that *T.vaporariorum* has the potential to evolve resistance when exposed to biocontrol using *E. formosa*, but whether this occurs in practice will depend on the evolutionary potential of the parasitoid and the costs of resistance in host.

In our first experiment, we show that whitefly (*T. vaporariorum*) populations harbor sufficient heritable variation in resistance to enable an evolutionary response to parasitism by *E. formosa*. Our estimates of heritability for resistance suggest a genetic basis for this trait. In our second experiment, we identified life history costs of *E. formosa* attack: whiteflies that survived parasitism had reduced fecundity and lower egg hatching success than unexposed controls. Life-history trade-offs are recognized as a key constraint on resistance evolution (Holt and Hochberg 1997) and may be particularly important for augmentative biocontrol, where agents are mass-reared and released without establishment (Stenberg et al. 2021). In contrast, coevolutionary dynamics may play a greater role in classical biocontrol, where long-term establishment is expected.

### Mechanisms of Resistance

Insects resist parasitoids through diverse mechanisms, from protective endosymbionts (Vorburger 2022) to behavioral adaptations (escape/evade rather than resist; Zalucki and Furlong 2017). However, genetically encoded (“endogenous”) immune responses to parasitoid attack are perhaps the best understood at the genetic and cellular level (Hillyer 2016). When a parasitoid egg is laid inside the host a process called ‘encapsulation’ is triggered, whereby layers of immune cells form around the egg, trapping it and preventing further development (Sideri et al. 2008). The cellular immune response leaves a visible scar as the injury heals by melanisation (Brady and White 2012, see Supplementary Fig. S3).

A key observation that motivated this study was the presence of such melanisation marks on many *T. vaporariorum* nymphs exposed to *E. formosa* in our laboratory colonies. Despite clear evidence of having been stung, many of these nymphs survived to adulthood, indicating variation in resistance to parasitism. In Experiment 1, we measured overall survival after exposure to *Encarsia* (without scoring melanisation) and found that, on average, 30% of exposed nymphs survived. In Experiment 2, we recorded melanisation marks as a qualitative indicator of parasitism in order to separate stung and unstung individuals to assess life history costs (but did not quantify survival specifically for marked versus unmarked individuals). The presence of melanisation marks on surviving whitefly points the cellular immune response as a potential mechanism underlying resistance in this system, but studies which combine quantative genetics and immune assays [ie phenoloxidase (PO) activity in the hemolymph, ie Cotter and Wilson 2002] are needed to explicitly test this.

### Costs of Resistance

Our study revealed fitness costs linked to resistance in *T. vaporariorum*, in particular lower fecundity and reduced hatch rates. Similar costs of have been demonstrated in other host–parasitoid systems (Takigahira et al. 2014) including in endosymbiont-mediated resistance in aphids (ie Gwynn et al. 2005, Takigahira et al. 2014, Jamin and Vorburger 2019, Käch et al. 2018). However, unlike aphid studies where resistant and susceptible lines can be compared directly (eg by curing symbionts, using naturally endosymbiont free lines), we could only measure the costs incurred after resisting parasitism, not the baseline costs of carrying resistance alleles in the absence of attack. In aphids, endosymbiont status acts as a phenotypic/genotypic “marker” of resistance, allowing researchers to maintain and compare resistant and susceptible lines under identical, parasite-free conditions. No equivalent marker exists for *T. vaporariorum*, so we cannot distinguish resistant individuals in the absence of parasitism to measure the baseline fitness costs of carrying resistance alleles. As a result, our estimates here reflect only the costs incurred after resisting parasitism, rather than the underlying costs of the resistance phenotype itself.

We recommend that future studies focus on developing markers to identify resistant *T. vaporariorum* using in-depth phenotypic and genomic screening, ideally within a full-sib/half-sib quantitative genetic framework. The recent chromosome-level assembly of the *T. vaporariorum* genome (Xie et al. 2020) opens up additional possibilities for developing molecular markers of resistance and integrating genomic tools with quantitative genetic approaches, greatly enhancing the scope for future studies. Such studies would be invaluable for a number of lines of enquiry. They would (1) make it possible to identify and maintain resistant and susceptible lines for direct costs-of-resistance assays, (2) provide more accurate estimates of heritability and the magnitude and source of maternal effects, and (3) generate data for robust predictive modeling of resistance evolution and viable intervention strategies for *T. vaporariorum*.

### Applications

Our results add to the small but growing number of case studies that demonstrate resistance to parasitoid-based biocontrol can evolve under certain conditions. Based on our results and general patterns of resistance observed in other systems we suggest two possible strategies that could help to slow the spread of resistance: (1) Exploiting costs of resistance to maintain susceptible pest genotypes. In the absence of parasitism, life history costs should maintain resistance alleles at low frequencies, slowing the spread of resistance traits so that biocontrol agents that are reared for augmentative release remain effective in the long-term. Strategies that increase spatial and temporal heterogeneity can amplify these trade-offs, for instance, additional stressors (eg suboptimal host plants, long dispersal distances between favored host plants) disadvantage resistant individuals and weaken directional selection (Mangan et al. 2023). This logic mirrors refuge strategies used in managing resistance to transgenic crops (Alstad and Andow 1995) and is supported by empirical studies and models (ie Ives et al. 2020).

(2) Enhancing the genetic diversity and performance of biocontrol agents is another strategy to mitigate resistance evolution (Leung et al. 2020). Traditionally, this has relied on ad-hoc genetic rescue, where wild-type individuals are introduced into mass-reared cultures to reduce inbreeding depression and boost reproductive success (Rasmussen et al. 2018). Genetic rescue could also improve biocontrol efficacy if alleles co-adapted under resistance evolution are introgressed into commercial populations. More targeted approaches are now emerging though, integrating quantitative genetics and genomics into biocontrol design (Leung et al. 2020). Artificial selection on beneficial traits, such as parasitoid virulence, has shown promise in theory (Chattington et al. 2025), and high heritability estimates for some complex traits support this potential (Chattington et al. 2025). In *E. formosa*, artificial selection is challenging due to parthenogenetic reproduction, but studies have revealed heritable variation in parasitism success across 3 lines on *Bemisia tabaci* (Henter and van Lenteren 1996). Although such variation has not yet been demonstrated for *T. vaporariorum*, screening across multiple lines could uncover genotypes with greater virulence. If identified, the asexual life history of *E. formosa* could actually be advantageous, helping preserve beneficial genetic variation by limiting drift and adaptation to laboratory conditions (de Paula et al. 2023).

Our study demonstrates that there is variation in resistance to the parasitoid *E. formosa* in the glasshouse whitefly *T. vaporariorum* and this variation appears to be both heritable and associated with significant life history costs. Our findings add to the growing number of cases which show resistance to parasitoid-based biocontrol can evolve (Holt and Hochberg 1997). However, the fitness costs that resistant *T. vaporariorum* nymphs experience later in life may provide a lifeline, constraining the spread of resistance under many ecological scenarios. This finding reinforces the view that, compared to chemical insecticides, biocontrol is evolutionarily stable and resilient to resistance evolution, particularly when pragmatic management strategies leverage these trade-offs.

## Supplementary Material

toaf338_Supplementary_Data

## Data Availability

All data, code, and supplementary materials are available on the of website https://osf.io/axbr5/ (doi: 10.17605/OSF.IO/AXBR5).
