# Supplementary material for: Is resistance futile? Life-history costs of escaping parasitoid attack in a major crop pest
Source: J Econ Entomol. 2025 Dec 5;119(2):823–30. doi: 10.1093/jee/toaf338 (PMC13075820; doi:10.1093/jee/toaf338)
Supplement: toaf338_Supplementary_Data [file toaf338_supplementary_data.docx]

Supplementary material


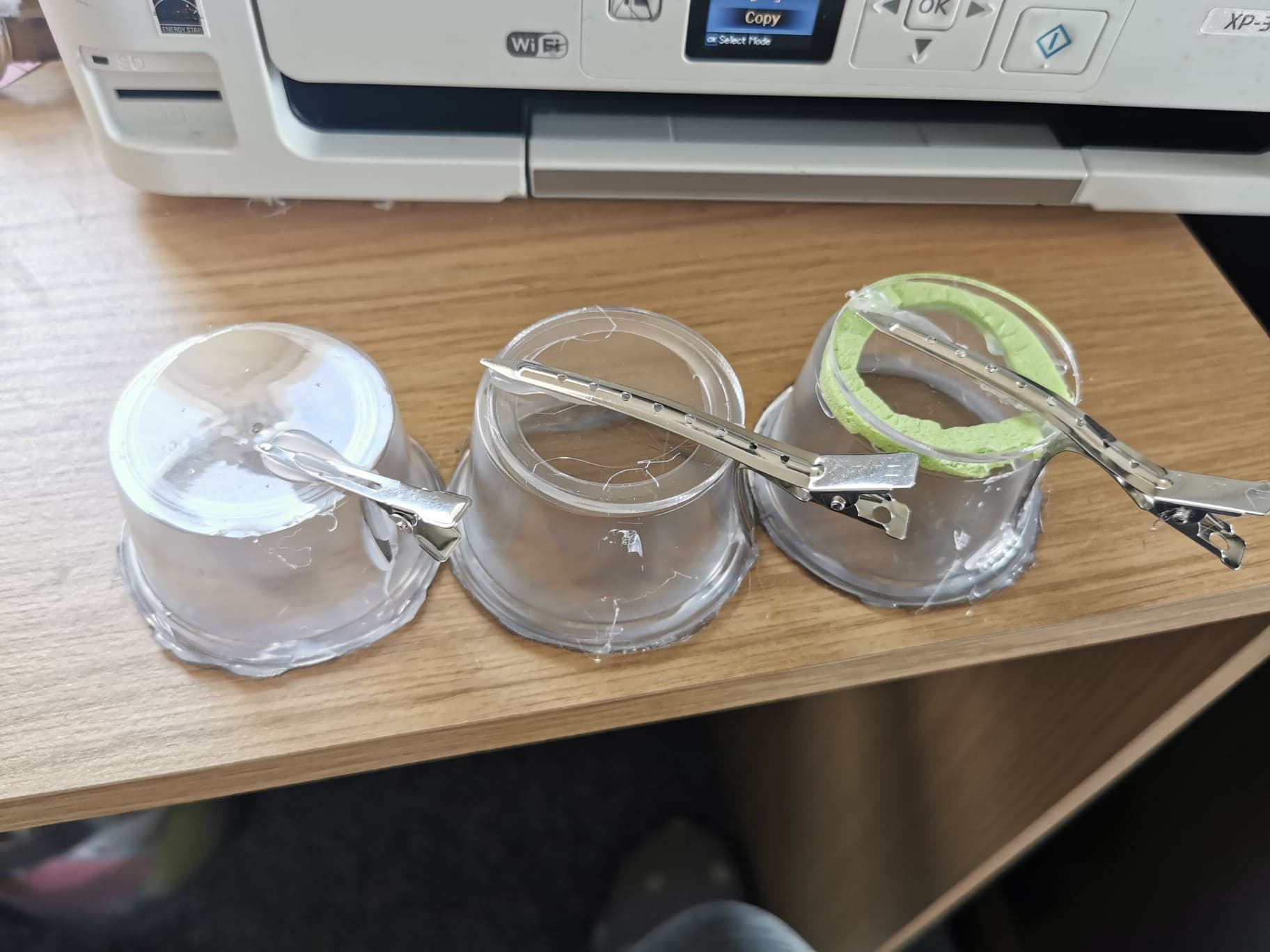

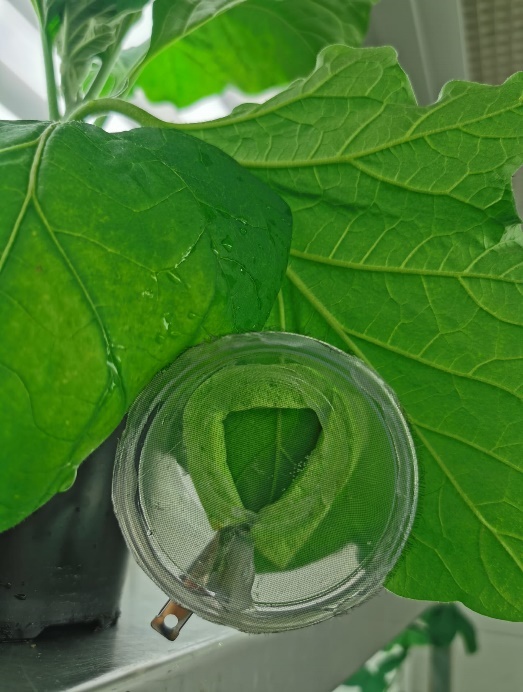

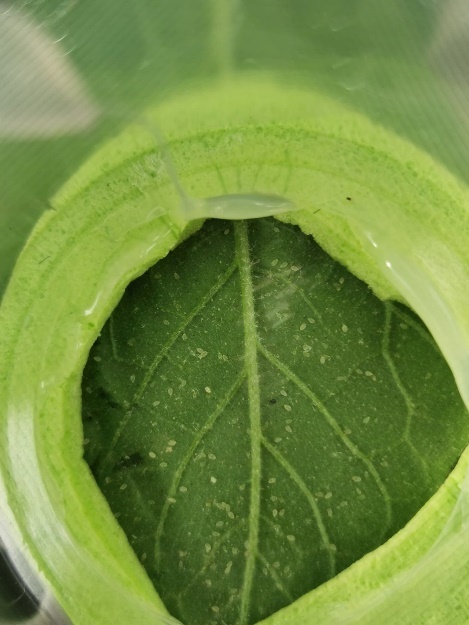


**Figure 1:** A) clip cage design; B) clip cage attached to an aubergine leaf; C) second/ third instar nymphs on a leaf inside the clip cage.

**Supplementary methods S2**


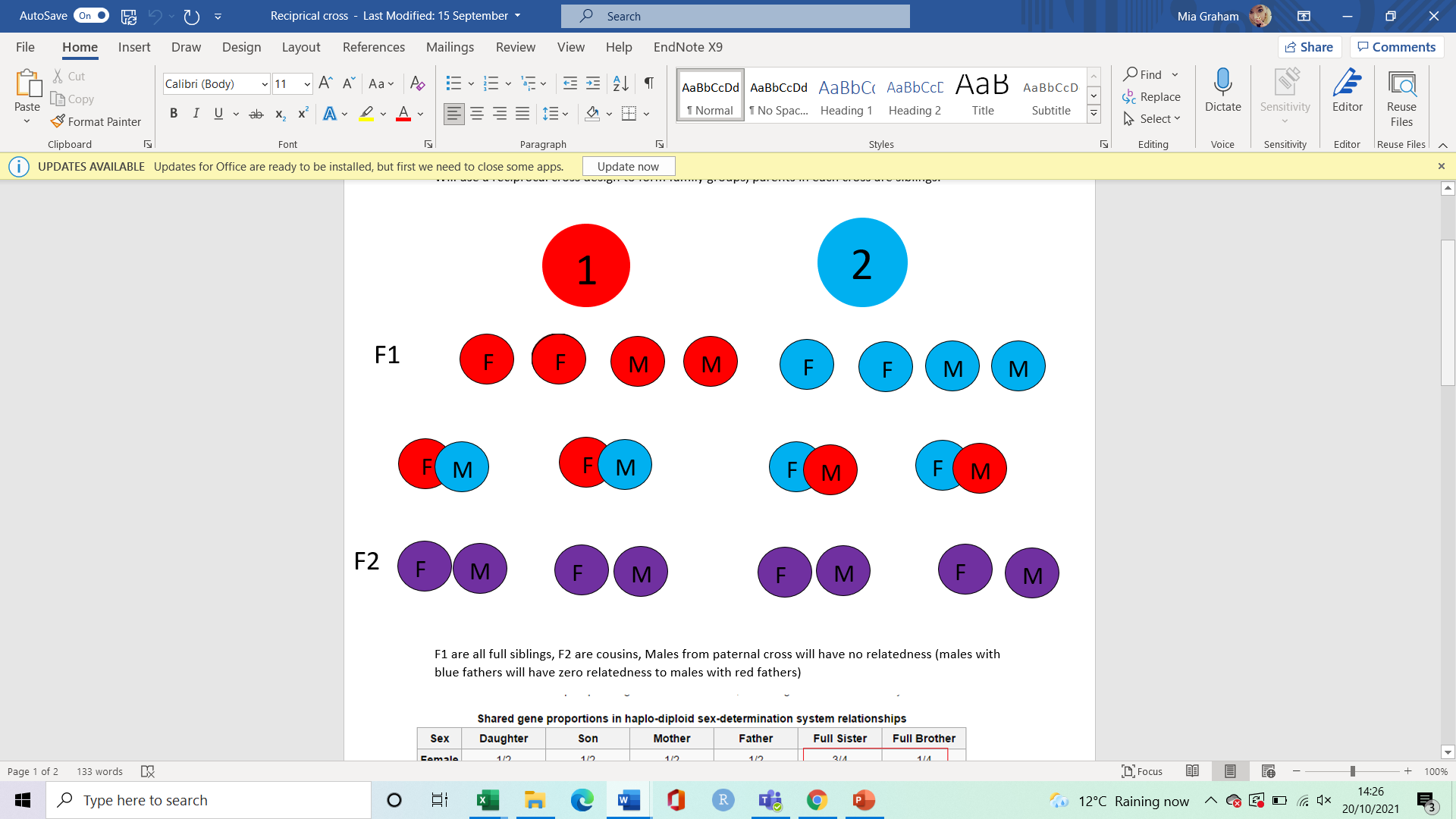


**Figure 2:** The reciprocal crossing scheme used to establish the 35 whitefly lines for the experiment crossed offspring of two independent whitefly lines, crossing females and males in both directions to determine if maternal effects alter offspring parasitism. Red circles indicate one line, the blue circles a second whitefly line. F1 and F2 indicate the first and second generations of offspring. Females from line one are mated with males of line two and vice versa. The purple circles show the offspring as a result of breeding line 1 with line 2. This scheme occurred for all 35 lines.

**Table 1:** the reciprocal crossing scheme used to generate families. Initial founding populations were given an ID, where males from one population were crossed with females from a second population in both directions to give a Line ID, e.g females from population 30 were crossed with males from population 33, and females from population 30 were crossed with males from population 33, these were all given a Line ID of A.

| Replicate ID | Line ID | Female adult ID | Male adult ID | Plant ID | Treatment |
| --- | --- | --- | --- | --- | --- |
| A3033.1 | A | 30 | 33 | A | Control |
| A3330.1 | A | 33 | 30 | L | Wasp |
| AA2934.3 | AA | 34 | 29 | J | Control |
| AA2934.3 | AA | 34 | 29 | P | Control |
| AA2934.1 | AA | 29 | 34 | O | Wasp |
| AB1448.2 | AB | 14 | 48 | P | Control |
| AB4814.1 | AB | 48 | 14 | R | Wasp |
| AC26.2 | AC | 2 | 6 | J | Control |
| AC26.2 | AC | 2 | 6 | O | Wasp |
| AC26.3 | AC | 2 | 6 | R | Wasp |
| AD4711.3 | AD | 47 | 11 | J | Control |
| AD1147.1 | AD | 11 | 47 | L | Wasp |
| AE623.2 | AE | 62 | 3 | J | Control |
| AE623.2 | AE | 62 | 3 | O | Wasp |
| AF3582.1 | AF | 35 | 82 | K | Control |
| AF3582.2 | AF | 35 | 82 | L | Wasp |
| AG2075.1 | AG | 20 | 75 | K | Control |
| AG2075.2 | AG | 20 | 75 | L | Wasp |
| AH6145.1 | AH | 61 | 45 | K | Control |
| AH6145.2 | AH | 61 | 45 | L | Wasp |
| AJ6668.1 | AJ | 66 | 68 | AH | Control |
| AJ6668.1 | AJ | 66 | 68 | X | Wasp |
| AJ6668.2 | AJ | 66 | 68 | Y | Wasp |
| AK7011.1 | AK | 70 | 11 | AH | Control |
| AK7011.1 | AK | 70 | 11 | X | Wasp |
| AK7011.2 | AK | 70 | 11 | Y | Wasp |
| AL6487.1 | AL | 64 | 87 | AA | Control |
| AL8764.1 | AL | 87 | 64 | AH | Control |
| AL8764.2 | AL | 87 | 64 | Y | Wasp |
| B82.1 | B | 8 | 2 | A | Control |
| B82.3 | B | 8 | 2 | L | Wasp |
| C510.1 | C | 5 | 10 | A | Control |
| C105.1 | C | 10 | 5 | I | Wasp |
| D2513.3 | D | 25 | 13 | H | Control |
| D2513.2 | D | 25 | 13 | B | Wasp |
| E732.1 | E | 7 | 32 | G | Control |
| E732.3 | E | 7 | 32 | I | Wasp |
| F1742.1 | F | 17 | 42 | G | Control |
| F4217.1 | F | 42 | 17 | H | Control |
| F4217.2 | F | 42 | 17 | I | Wasp |
| G1214.1 | G | 12 | 14 | K | Control |
| G1214.1 | G | 12 | 14 | P | Wasp |
| H1829.1 | H | 18 | 29 | G | Control |
| H2918.2 | H | 29 | 18 | P | Wasp |
| I2734.1 | I | 27 | 34 | G | Control |
| I2734.2 | I | 27 | 34 | H | Control |
| I2734.2 | I | 27 | 34 | P | Wasp |
| I3427.1 | I | 34 | 27 | I | Wasp |
| J938.1 | J | 9 | 38 | H | Control |
| J389.1 | J | 38 | 9 | G | Control |
| J938.2 | J | 9 | 38 | I | Wasp |
| K1920.1 | K | 19 | 20 | G | Control |
| K1920.3 | K | 19 | 20 | I | Wasp |
| L374.2 | L | 37 | 4 | E | Control |
| L437.2 | L | 4 | 37 | D | Wasp |
| L374.1 | L | 37 | 4 | A | Wasp |
| M3849.1 | M | 38 | 49 | E | Control |
| M4938.1 | M | 49 | 38 | A | Wasp |
| N4650.2 | N | 46 | 50 | G | Control |
| N5046.2 | N | 50 | 46 | E | Control |
| N5046.1 | N | 50 | 46 | D | Wasp |
| O229.1 | O | 2 | 29 | G | Control |
| O229.2 | O | 2 | 29 | H | Wasp |
| P313.1 | P | 31 | 3 | G | Control |
| P313.2 | P | 31 | 3 | H | Wasp |
| R4017.2 | R | 40 | 17 | K | Control |
| R1740.1 | R | 17 | 40 | L | Wasp |
| S4518.1 | S | 45 | 18 | N | Control |
| S1845.1 | S | 18 | 45 | Q | Wasp |
| U418.1 | U | 41 | 8 | Q | Control |
| U418.1 | U | 41 | 8 | N | Wasp |
| V1654.3 | V | 16 | 54 | C | Control |
| V1654.3 | V | 16 | 54 | U | Wasp |
| V5416.1 | V | 54 | 16 | D | Wasp |
| W3860.1 | W | 38 | 60 | K | Control |
| W6038.3 | W | 60 | 38 | C | Control |
| W3860.3 | W | 38 | 60 | W | Wasp |
| W6038.3 | W | 60 | 38 | U | Wasp |
| X751.3 | X | 7 | 51 | C | Control |
| X751.5 | X | 7 | 51 | E | Control |
| X751.4 | X | 7 | 51 | D | Wasp |
| X751.5 | X | 7 | 51 | W | Wasp |
| Y2550.1 | Y | 25 | 50 | C | Control |
| Y2550.2 | Y | 25 | 50 | K | Control |
| Y2550.2 | Y | 25 | 50 | D | Wasp |
| Y5025.1 | Y | 50 | 25 | W | Wasp |
| Z238.3 | Z | 23 | 8 | E | Control |
| Z823.1 | Z | 8 | 23 | W | Wasp |


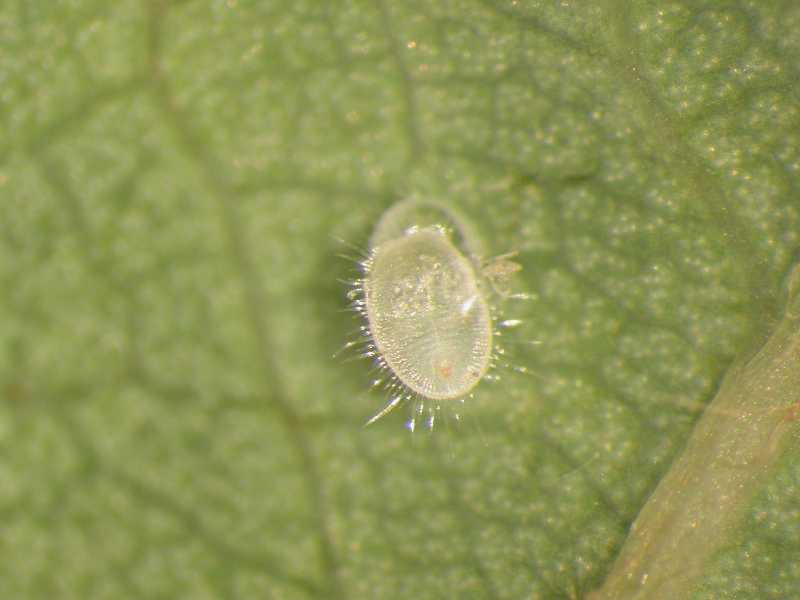

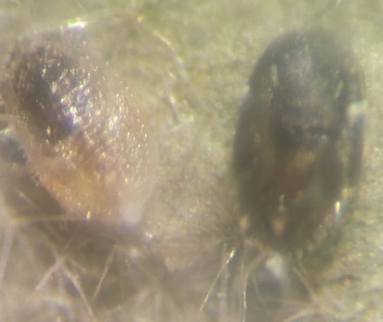

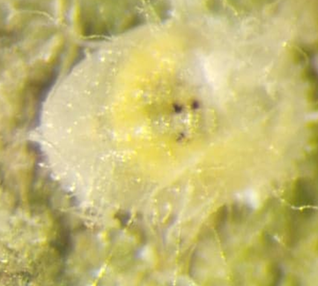


C

B

A

**Figure 3:** A) A unparasitised whitefly pupae; B) A whitefly nymph with three melanisation marks from positive parasitation by *E. formosa*; C) a parasitised whitefly nymph.

**Supplementary methods 4 (estimating heritability)**

We estimated heritability for survival following parasitism by running a linear mixed-effects model where the response variable was the proportion of dead individuals at 10 days post-exposure. The model was run separately for the Encarsia and control treatments, and included random effects of dam (F), sire (M), block, and plant to partition genetic and environmental variance components using restricted maximum likelihood (REML). We extracted variance components following the approach outlined in Hansen et al. (2024), who demonstrate how to derive between-sire, between-dam, and residual variance components from random-effects models fitted to single-generation sib designs.

The variance components extracted from the model for the Encarsia treated wasps was as follows:

Var_sire = 0.004546

Var_dam = 0.015060

Var_plant = 0.002291

Var_block = 0.003061

Var_residual = 0.008251

Because Trialeurodes vaporariorum is a haplodiploid species (males are haploid, females are diploid), we followed the quantitative genetic framework for haplodiploid organisms developed by Liu & Smith (2000). In this framework:

- Variance among sires (haploid males) reflects half of the additive genetic variance (VA):

Var_sire = VA/2

Rearrange to:
VA = 2 × Var_sire = 2 × 0.004546 = 0.009092

- Variance among dams (diploid females) includes additive (VA) and dominance variance (VD) as well as common family variance (VEc - which we assume is captured by Var_plant + Var_block).
 Var_dam = VA/4 + VD/2 + VEc

Rearranging (and assuming VEc = Var_plant + Var_block = 0.002291 + 0.003061 = 0.005352):
 VD = 2 × (Var_dam – (VA/4) – VEc)

VD = (0.015060 - (0.009092/4) – 0.005352) = 0.01487

- Environmental variance (Ve) was taken as the residual variance in the model (Var_resid) as Var_plant and Var_block were included in VEc.
 Ve = Var_resid = 0.008251

- Total phenotypic variance was therefore:
 VP = VA + VD + Ve = 0.009092 + 0.01487 + 0.008251 = 0.0322

- We estimated haplodiploid-adjusted narrow-sense heritability as:
 h² = VA / VP = 0.009092 / 0.0322 ≈ 0.2823

Note that the experimental design was not optimised for quantitative genetic analysis (e.g. unbalanced families and incomplete plant nesting) and does not allow us to test the assumption of additive-dominance using a joint-scaling test (as we only assayed one generation). However, the model allows us to derive approximate heritability estimates to evaluate whether resistance to parasitism has a heritable genetic basis. Our estimate of heritability (h²) represents the narrow-sense heritability (i.e. the proportion of phenotypic variance attributable to additive genetic variance) adjusted for haplodiploid inheritance. It provides an approximate measure of the evolutionary potential for resistance to evolve via natural selection in this system.

**R Code for Haplodiploid Heritability Estimation**

model_Encarsia_random <- lmer(prop_dead ~ (1|F) + (1|M) + (1|Block) + (1|plant), data = E_only)

simulationOutput <- simulateResiduals(fittedModel = model_Encarsia_random, plot = T, use.u = T)

summary(model_Encarsia_random)

##haplodiploid heritability (modified from Liu & Smith)

# ----- Extract variance components from lmer -----

vc <- as.data.frame(VarCorr(model_Encarsia_random))

# Helper to pull a variance by grouping factor name

get_var <- function(grp, vc_df) {

x <- vc_df$vcov[vc_df$grp == grp]

if (length(x) == 0) return(0) else return(x[1])

}

Vsire <- get_var("M", vc) # variance among sires (haploid males)

Vdam <- get_var("F", vc) # variance among dams (diploid females)

Vplant <- get_var("plant", vc) # plant variance

Vblock <- get_var("Block", vc) # block variance

Vresid <- get_var("Residual", vc) # residual variance

# Environmental variance

Ve <- Vresid

# Total variance due to shared environment within families

VEc <- Vplant + Vblock

# ----- Haplodiploid-adjusted components (Liu & Smith 2000) -----

# Var_sire = 1/2 * VA -> VA = 2 * Var_sire

# Var_dam = 1/4 * VA + 1/2 * VD + VEc

haplo_components <- function(Vsire, Vdam, Vplant, Vblock, Vresid, VEc) {

VA <- 2 * Vsire

Ve <- Vresid

VD <- 2 * (Vdam - (VA/4) - VEc)

VP <- VA + VD + Ve

h2 <- VA / VP # narrow-sense heritability (additive only), haplodiploid-adjusted

list(VA = VA, VD = VD, Ve = Ve, VP = VP, h2 = h2,

inputs = list(Vsire = Vsire, Vdam = Vdam, Vplant = Vplant,

Vblock = Vblock, Vresid = Vresid, VEc = VEc))

}

# Compute components and h2

res_haplo <- haplo_components(Vsire, Vdam, Vplant, Vblock, Vresid, VEc)

print(res_haplo)

cat(sprintf("Haplodiploid-adjusted h2 = %.3f\n", res_haplo$h2))


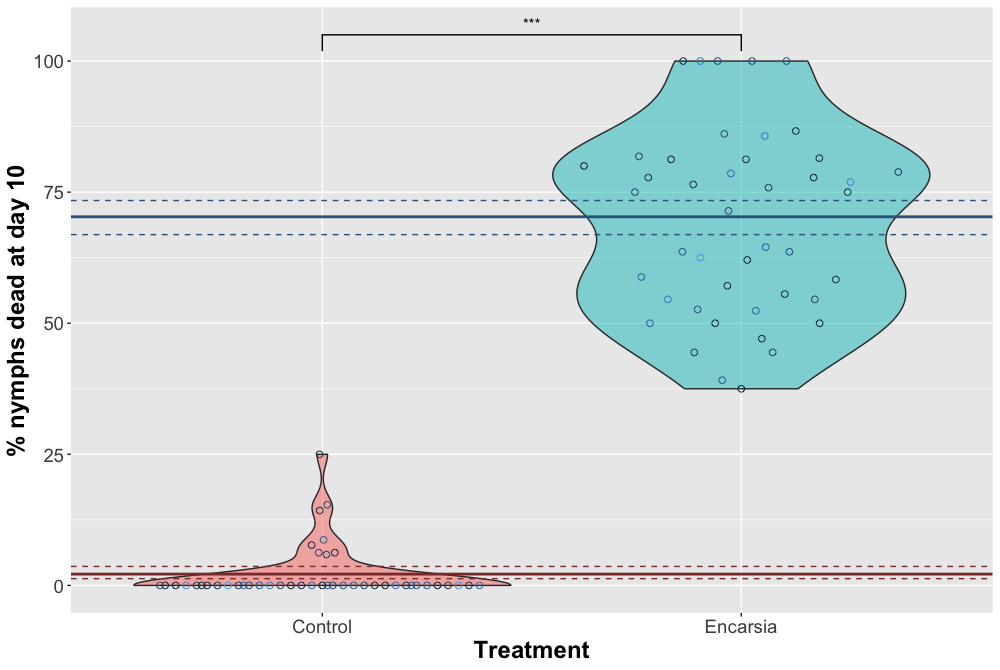


**Figure 4:** Percentage % of *T. vaporariorum* nymphs which were dead 10 days after being exposed to the parasitoid wasp *Encarsia formosa* (Encarsia) or not (Control). Solid and dashed lines represent overall binomial mean ± binomial CI in the Control (bottom, red) and *Encarsia* treated (top, blue) whitefly. Survival was higher in the control whitefly compared to the *Encarsia* treated; significant differences are indicated by a * (<0.05) over the bars. Points on each plot are shaded by sire ID.


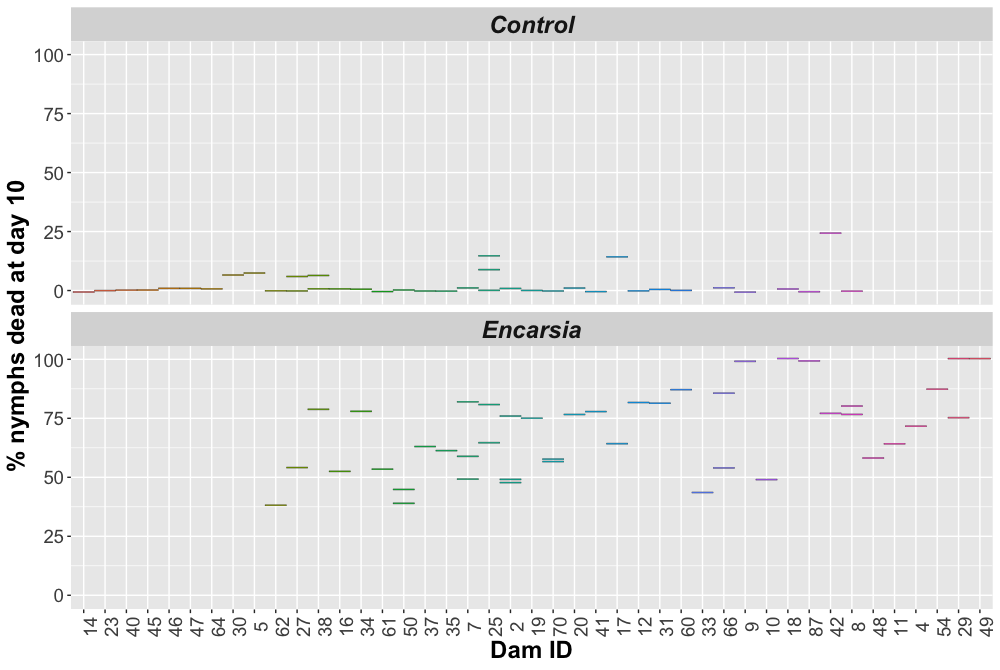


**Figure 5:** Percentage % of *T. vaporariorum* nymphs which were dead10 days after being exposed to the parasitoid wasp *Encarsia formosa* (Encarsia) or not (Control) by maternal (Dam) ID. Note that offspring for some mothers only appear in one treatment condition and replicates for each dam are not balanced.


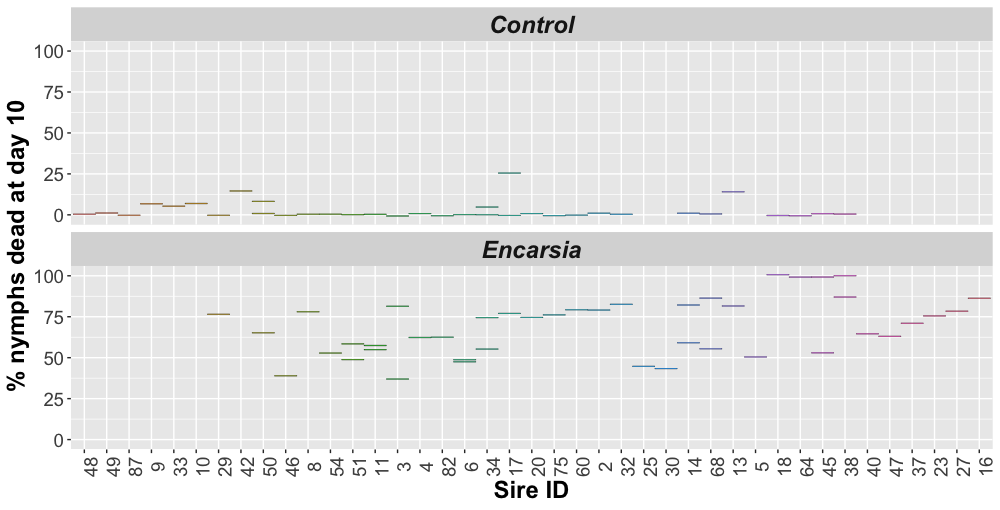


**Figure 6**: Percentage % of *T. vaporariorum* nymphs which were dead10 days after being exposed to the parasitoid wasp *Encarsia formosa* (Encarsia) or not (Control) by paternal (sire) ID. Note that offspring for some fathers only appear in one treatment condition and replicates for each sire are not balanced.
